# Comparative study of qualitative and quantitative methods to determine toxicity level of *Aspergillus flavus* isolates in maize

**DOI:** 10.1371/journal.pone.0189760

**Published:** 2017-12-15

**Authors:** Meena Shekhar, Nirupma Singh, Ram Dutta, Shrvan Kumar, Vinay Mahajan

**Affiliations:** 1 ICAR-Indian Institute of Maize Research (IIMR), Pusa Campus, New Delhi, India; 2 ICAR-Directorate of Groundnut Research (DGR), Junagadh, Gujarat, India; Tallinn University of Technology, ESTONIA

## Abstract

An attempt was made to compare between easy and inexpensive qualitative method (ammonia vapour test) and analytical methods (thin layer chromatography and enzyme-linked immunosorbent assay) for identification of aflatoxigenic isolates of *Aspergillus flavus* in maize. In this comparative study the toxicity level of *A*. *flavus* isolates exhibited 100% agreement among ammonia vapour test, ELISA and TLC for highly toxigenic (>2000 ppb) and toxigenic (501–2000 ppb) isolates while 88.5% agreement observed for least toxic (<20 ppb) isolates. In ammonia vapour test 51% of *A*. *flavus* isolates showed creamish or no colour change corresponding to least toxic/atoxic (<20ppb) category estimated by ELISA. Similarly 22% highly toxic isolates exhibited plum red colour, 12% moderately toxic indicated pink colour and 10% toxic isolates showed red colour. However, 11.5% isolates were found to be false positive in cream colour category (least toxic) and 28.5% false negatives in pink colour (moderately toxic) category. The isolates from different agroclimatic zones of maize in India showed high variability for aflatoxin B_1_ (AFB_1_) production potential ranging from 0.214–8116.61 ppb. Toxigenic potential of *Aspergillus flavus* isolates in culture was further validated by inoculating maize grain sample with four different isolates with varied toxin producing ability. With good agreement percentage between cultural and analytical methods the study concludes the ammonia vapour test to be easy, inexpensive, reliable and time saving method that can be used for segregating or pre-screening of contaminated samples from bulk food/feed stock.

## Introduction

Aflatoxins are naturally occurring mycotoxin and outrageous contaminants of the important agricultural commodities such as corn, peanuts, pistachio, Brazil nuts, oilseeds like cottonseed and copra [1, 2, 3].Exposure to aflatoxins causes both acute and chronic risks to lower income populations in tropics mainly consuming large quantity of maize or groundnut as staples. Consumption of highly aflatoxin contaminated food leads to liver failure within 1–2 weeks, known as acute aflatoxicosis. Aflatoxicosis may lead to cancer and immune suppression and in acute condition may cause death [4]. In India, during 1974 an outbreak of hepatitis caused many deaths attributed to the consumption of heavily aflatoxins contaminated maize [5]. It is well established that chronic exposure to aflatoxins leads to liver cancer (especially where hepatitis is prevalent), and this is estimated to cause as many as 26,000 deaths annually in Africa and south of the Sahara. Aflatoxin contamination is one of the most challenging and serious food safety problem worldwide, to establish control measures extensive research work is being done in this area. Growth of commercial markets for food and feed, including exports is affected by aflatoxin contamination [6].

Aflatoxins are polyketide-derived hepato-carcinogenic and mutagenic secondary metabolites, produced by *Aspergillus* spp. [7]. It contains about twenty similar compounds belonging to a group called difurano-coumarins, and only four (B1, B_2_, G_1_ and G_2_)are found naturally in foods. Of these, Aflatoxin B_1_ is the most toxic and commonly found toxin which on extreme exposure causes acute toxicity in mammals, birds and fish as well as in humans.

Aflatoxins are produced by fungi of genus *Aspergillus*, especially *A*. *flavus*, *A*. *parasiticus*, *A*. *nomius* and *A*. *pseudotamrii* [8]. *Aspergillus flavus* is predominant in maize [9, 10]. The warm and humid favours *Aspergillus flavus* for fungal colonization and cob rot in maize [11]. Extended storage of maize in poor storage conditions enhances fungal growth which promotes the production of mycotoxins [12]. Aflatoxin-producing potential of *A*. *flavus* isolates is highly variable ranging from high aflatoxin production to completely low/nontoxic. Thus, the severity of spoilage of food and feed owing to aflatoxin contamination depends on the concentration of AFB_1_ produced by isolates [13, 14, 15, 16, 17].

Many highly specific and sensitive methods for estimation of aflatoxin in commodities are there such as high performance liquid chromatography (HPLC), enzyme linked immuno- sorbent assay (ELISA), thin layer chromatography (TLC) and fluorescence polarization assay *etc*. [18].These analytical methods have been proven to be accurate and reliable, but are costly and require large quantities of expensive organics. Chromatographic methods are automated and highly accurate method to identify certain chemical components in a sample, but it can be costly, complex and work for one sample at a time. Now a day, commercially available ELISA kits are easy for quantifying total aflatoxin concentration but it doesn’t work for identifying individual aflatoxin component (B_1_, B_2_).

Demand for monitoring aflatoxin has increased in developing countries due to high incidence of liver and other cancers [19]. The recurring cost in analytical methods is expensive, however, the qualitative methods provide a cheaper option to screen large number of aflatoxin contaminated samples. Among the many qualitative methods, visual indictors are thought to be highly predictive for aflatoxin build up in agricultural commodities. However, exposure to ammonia vapours can detect the aflatoxin production by changing the colour of toxigenic colonies from yellow to pink [20].

In the present investigation the accuracy of qualitative method (ammonia vapour test) was ascertained by comparing with analytical (TLC and ELISA) methods. For the study, maize grain samples collected from farmers field of four agro-climatic zones of India. Sixty isolates collected from the samples, purified and characterized on the basis of toxigenic potential by using qualitative (ammonia vapour test) and quantitative (ELISA & TLC) methods. The culture results were further validated on maize grains (*in vivo*).

## Materials and methods

Sixty maize grain samples were collected from four agroclimatic zones of India- Himachal Pradesh (Zone I—North Hill Zone), Haryana and Delhi (Zone II- North Western Plain Zone), Bihar (Zone III- North Eastern Plain Zone), and Andhra Pradesh (Zone IV- Peninsular Zone). No specific permission was required to select location for the study. The field study did not involve endangered or protected species. Sampling was done directly from farmers’ field with the sample size of 2 kg.

### Isolation and identification of fungal isolates

Ten kernels from each sample were surface sterilized and placed on culture plates containing Potato Dextrose Agar (PDA) medium (Hi Media Laboratories, Mumbai, India) and incubated at 28°C ±2°C for 3 days. *Aspergillus flavus* isolates were identified based on macroscopic and microscopic characteristics [21] and transferred on to agar slant. The stock culture of the isolates was maintained by transferring them on PDA plates and single colonies were picked up and transferred to culture plate for further study.

### Qualitative method for screening of *A*. *flavus* isolates for aflatoxin production

#### Ammonia vapour test

The isolates of *A*. *flavus* were cultured on PDA and incubated at 28°C ±2°C for 7 days. Two replications were maintained. After incubation, petri-dishes were turned upside down and 2 ml of concentrated ammonia solution (SRL Extra pure AR Grade) was poured into the lid of inverted culture plate and kept for 10–15 minutes to release ammonia vapour [20]. On exposure of culture to ammonia vapour, the colour development was recorded.

### Quantitative analysis of aflatoxin

#### Thin layer chromatography

**Fig 1:**
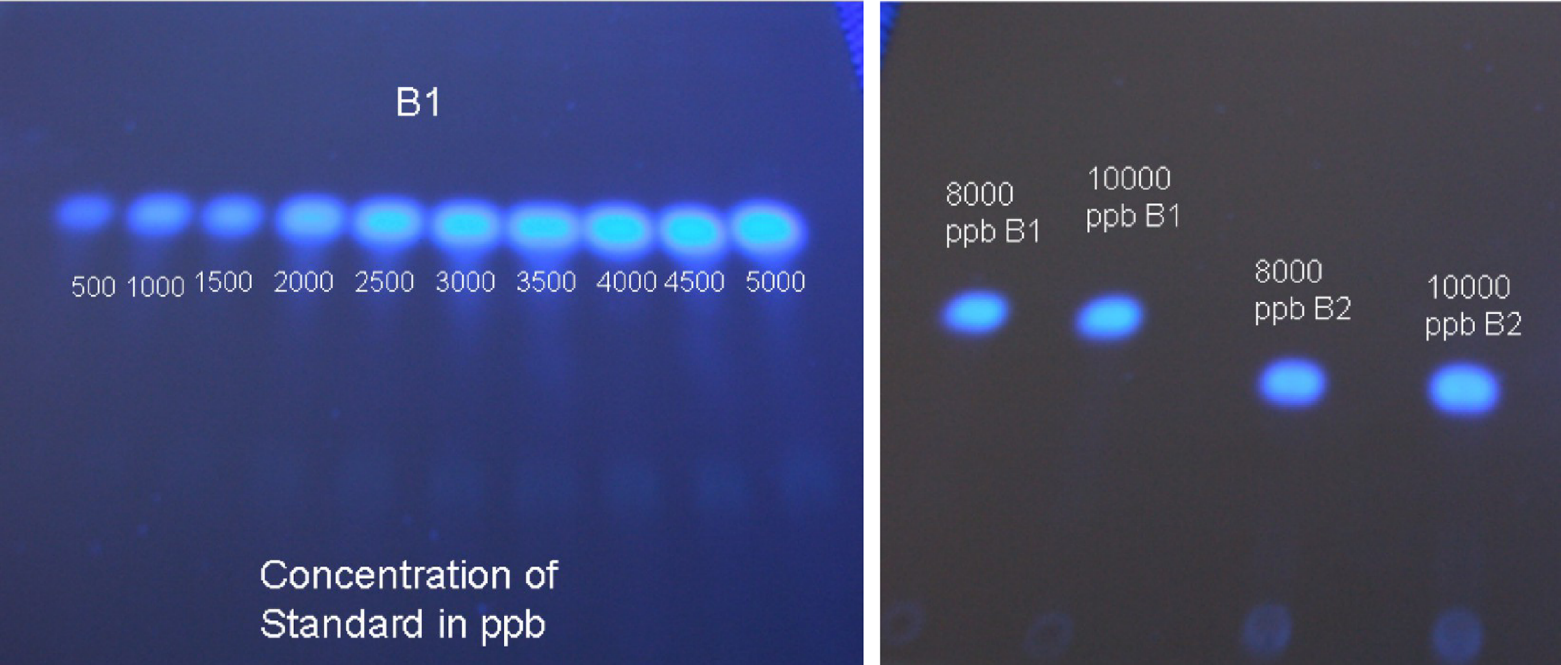
Spots of Aflatoxin on TLC plate. (A) Various concentrations of AFB_1_ standard (B) Confirmation of AFB_1_ and AFB_2_ spots.

Potato dextrose broth (HiMedia Laboratories, Mumbai, India) was prepared in 50 ml flask. Two sets were maintained for each isolate. Two mm bits of 1- week old culture of *A*. *flavus* was used to inoculate broth and incubated for ten days at 27°C in the dark. Cultures in broth were filtered through Whatman filter paper No. 41 and extracted with chloroform (25:25 v/v) in separating funnels. The bottom aqueous layer of chloroform was passed through an anhydrous sodium sulphate column to dry the remnant chloroform layer containing dissolved mycotoxins. The eluted solution/chloroform evaporated at 60°C in a vacuum evaporator. The residue was dissolved in 500 μl (0.5 ml) acetonitrile. Confirmation of AFB_1_ and B_2_ was done by TLC (Fig 1) using a slightly modified Association of Analytical Communities (AOAC) technique [11]. Aliquots of the acetonitrile extract (20 μl) was spotted in duplicate on TLC silica gel plate (Merck 60 F_254_), and was pre activated at 100°C for 25–30 minutes in oven. These TLC plates were developed in chloroform acetone mixture (9:1) and analyzed under UV light.

#### Enzyme-linked immunosorbent assay (ELISA)

Individual spots of Aflatoxin B_1_ were scrapped and dissolved in 1 ml acetonitrile for estimation of aflatoxin.Quantitative analysis of AFB_1_ was accomplished by indirect competitive ELISA [22]. Coating of microtitre plates (Nunc-Immuno^™^MicroWell^™^ 96 well) was done with AFB_1_–BSA in carbonate buffer (pH 9.6), which was left overnight at 4°C, and washed thrice with Phosphate-buffered saline Tween (PBST). Then 0.2% BSA added; plates were kept for incubation at 37°C for 1 hour. AFB_1_ standards (Sigma, St. Louis, USA) serial dilution from 1000 ppb to 0.9 ppb was used. Antiserum diluted in PBST–BSA (1:6000) was added in each well and incubated for 1 hour at 37°C. Goat antirabbit immunoglobulins conjugate to alkaline phosphatase was added at a 1:4000 dilution before incubation at 37°C for 45–60 minutes followed by washing three times and absorbance was recorded at 405 nm with an ELISA plate reader (Thermo Multiscane EX). Standard curves were obtained by plotting log_10_ values of AFB_1_ dilutions at A405. The AFB_1_ (ppb) in sample was determined from the standard curves by plotting the aflatoxin concentration on the “X” axis and optical density values on the “Y” axis. To test recovery of AFB_1_ 20g healthy maize grain extract was mixed with pure AFB_1_ (Sigma, St. Louis, USA) to have concentrations from 0.9, 2.0, 3.9, 7.8, 15.6, 31.3, 62.5, 125.0, 250.0, 500.0 to 1000.0 ppb. The limit of detection of AFB_1_ was 0.02ng/ml.

### *In vivo* toxigenic behaviour of *A*. *flavus* isolates

**Fig 2:**
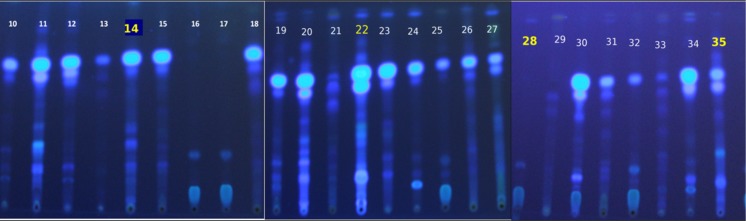
AFB_1_spots of different isolates with varying toxin producing potential [very high toxic (22), toxic (14), moderately toxic (35) and least toxic (28) isolates].

The toxigenic behaviour of *A*. *flavus* isolates expressed in cultures was further validated on healthy maize hybrid *i*.*e*.,Vivek QPM 9 under artificial inoculation condition. Four isolates with different toxin producing potential were selected for artificial inoculation *viz*., very high toxic (HT—*A*. *flavus* isolate No.22), toxic (T- *A*. *flavus* isolate No.14), moderately toxic (MT—*A*. *flavus* isolate No.35) and least toxic (LT—*A*. *flavus* isolate No. 28) (Fig 2). Spores from ten- day old *A*. *flavus* culture of isolates were harvested to prepare individual spore suspension in distilled water separately with concentration adjusted to 10^8^spores/ml. A quantity of 720g of maize hybrid was taken to maintain three replications for five treatments. From this, 120g of grains were used as initial *i*.*e*., without surface sterilized but washed in three changes of sterile distilled water. Rest 600g of grains were surface sterilized for 1 min in 2% NaOCl, washed in 3 changes of sterile distilled water. The surface sterilized grains were divided in 120g per treatment and filled separately in five flasks under aseptic condition. One flask was kept for check and rest four flasks were inoculated by each isolates respectively by dipping grains into spore suspension of *A*. *flavus* for ten minutes. After inoculation the grains of each treatment were divided into three replications (40g each). Each treatment/check incubated at temperature 26±2°C and relative humidity of 95±2% for 30 days. For estimation of AFB_1_ maize kernels from each sample were grounded and 20g powder mixed thoroughly with 70% methanol containing 0.5% KCI in a blender separately. This extract was allowed to shake for thirty minutes at 300 rpm and filtrated through Whatman filter paper No. 41and the extract obtained was used for indirect competitive ELISA.

### Statistical analysis

All of the statistical analyses were performed using SAS software (NC, USA), and the data were analyzed using ANOVA at the 95% confidence level. Results obtained by ELISA were categorized as “atoxigenic/least toxic” for isolates producing less than 20 ppb of aflatoxin in culture, “moderate toxic” for isolates producing between 20 and 500 ppb in culture, and “toxic” for isolates producing 500–2000 ppb and “highly toxic” for isolates producing >2000 ppb in culture. In each case, numbers of replications were 3 and mean with standard deviation of *in vivo* aflatoxin concentration were compared between control and aflatoxin categories.

## Result and discussion

### Identification of toxic *Aspergillus flavus* isolates using ammonia vapour test

**Fig 3:**
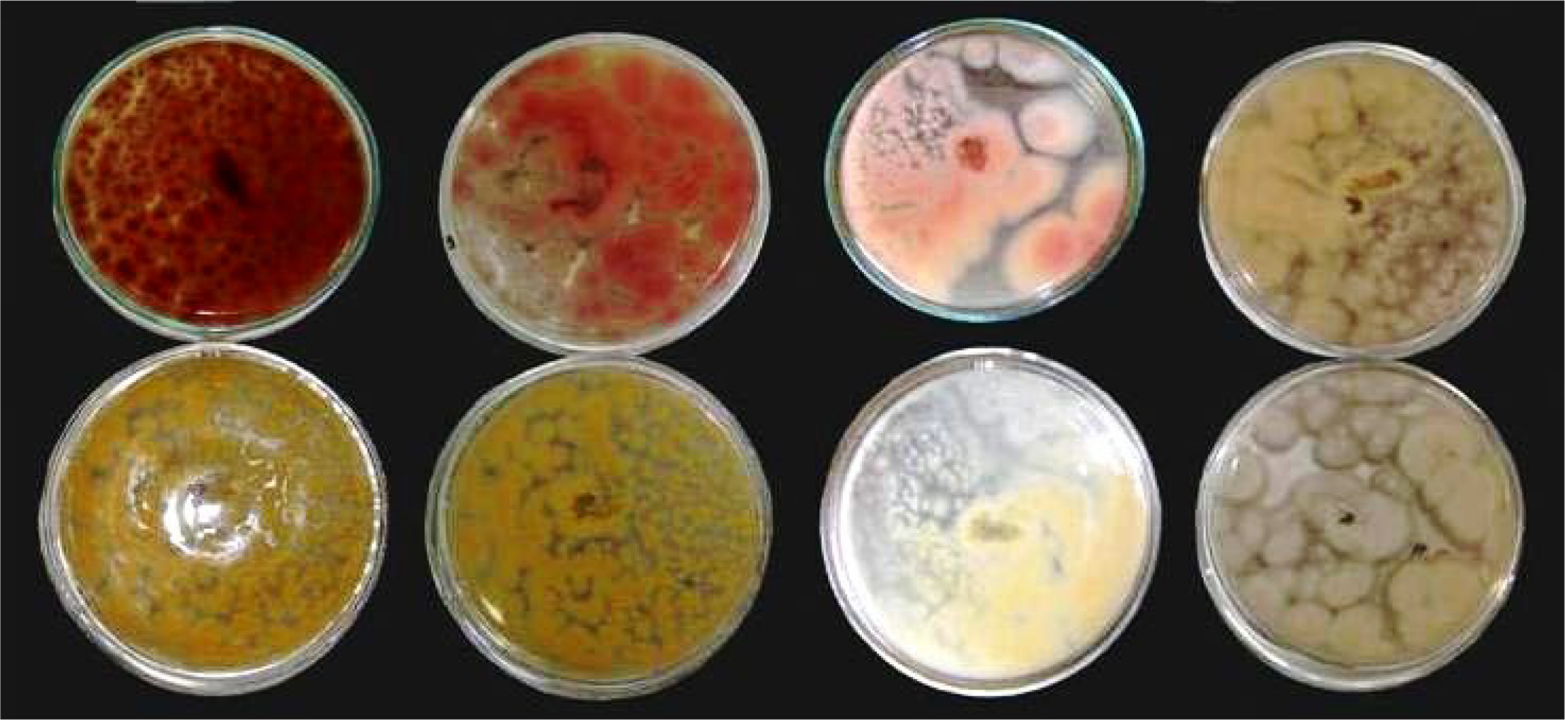
Appearance of various degrees of colours developed on *A*. *flavus* culture plates on exposure with ammonia vapour (Top: left to right Plum Red, Red, Pink, Cream; Bottom: left to right respective control).

Based on the cultural and physiological characteristics, fifty isolates were identified as *Aspergillus flavus* from maize grain samples using a taxonomic key and species descriptions by Klich 2002 [21]. These isolates exhibited variable toxin producing ability. In similar study by Hussein *et al*.,[23] it was found that isolates of *A*. *flavus* produced aflatoxins at variable rates when cultured under same conditions. In present study, on exposure of *A*. *flavus* cultures with ammonia vapour leads to varied degree of colour change, ranges from plum red, red and pink to cream. The colour of culture is correlated with the aflatoxin concentration estimated by ELISA as plum red being highly toxic (> 2000 ppb), red as toxic (501–2000 ppb), pink as moderate toxic (21–500 ppb) and cream colour as least toxic /non toxic (< 20 ppb)isolates(Fig 3). Colorimetric visualization showed the highest percentage of isolates (52%) exhibiting cream or no colour change. It was followed by 24% cultures turning to plum red colour, while 10% red colour and 14% to pink colour on exposing with ammonia vapour (Table 1). In this experiment, the concentration of AFB_1_ varied across the region ranging from 0.214 to 8116.61ppb as estimated by ELISA. Recently, in rice grain high level of variability was found and maximum isolates exhibited AFB_1_ concentration in range from 175 to 124 101 μg/kg [24]. In one of our recent study a high variability for aflatoxin production potential in maize was found among the isolates of *A*. *flavus* of different agro ecological zones of India. However no correlation was observed between genetic variability and toxin production potential of *A*. *flavus* isolates[25].The change in colour of the cultures as plum red, red, pink or cream colour on exposure of ammonia vapours is a useful and quick technique for identifying *A*. *flavus* isolates from highly toxic to least toxic. This method is inexpensive and less time taking for screening the large number of aflatoxigenic *Aspergillus flavus* isolates.

**Table 1:** Colour changes in *Aspergillus flavus* isolates after exposure to ammonia vapour and corresponding aflatoxin B_1_ levels.

	Colour change after Ammonia Vapour Test			
	Cream	Pink	Red	Plum red
**AFB**_**1**_^**a**^ **production (ppb)**	<20	21–500	501–2000	>2000
**Number of isolates**	26	7	5	12
**Percentage (%)**	52	14	10	24

^a^ Aflatoxin level detected by ELISA.

Limit of detection 0.02ng/ml.

### Comparison and agreement between aflatoxin assays

Screening of aflatoxin production potential in cultures of *A*. *flavus* isolates showed 52% of isolates with very low concentration of AFB_1_ (<20 ppb), 14% exhibited 21–500 ppb aflatoxin concentration,10% with 501– 2000ppb concentration and 24% detected with very high aflatoxin concentration above 2000 ppb (Table 1). The variability in aflatoxin production potential of *A*. *flavus* isolates is well documented in maize [26, 27], rice [28], pistachio [29] etc. The incidence of highly toxigenic isolates depends on genetic variability of *A*. *flavus* fungi, besides this, other factors like high temperature, crop rotation and history of crop cultivation also favour the occurrence of *A*. *flavus*. [30, 31, 32, 33, 34, 35]. With the quest for screening samples of food/feed contaminated with toxic isolates of *A*. *flavus* with ease and accuracy, the current work was undertaken to evaluate the cultural/ inexpensive method with high precision costly methods and simultaneously validate the results in maize grain samples.

Accuracy of cultural/Ammonia Vapour test was determined by comparing the concentration of AFB_1_ in each isolates by TLC and ELISA. The 22% of isolates giving plum red colour of very high toxic (>2000 ppb) category and 10% red colour isolates of highly toxic (501–2000 ppb) categories were 100% in agreement with ELISA and TLC analysis. The observation of pink colour on exposure with ammonia vapour had a 71.5% agreement rate and TLC had 85.7% agreement with ELISA in identifying moderate toxic (21–500 ppb) strains. As per the Food and Drug Administrative (FDA) guideline regulatory level, the permissible limit in maize for human consumption is 20 ppb.

Appearance of false positive in low frequency (11.5%) in least/nontoxic (<20 ppb) group, is a minor discrepancy observed between ELISA, TLC and ammonia vapour test with 88.5% agreement between cultural method and ELISA (Table 2). The false positive had more than 20 ppb of permissible limit of aflatoxin but less than 100 ppb concentration. However, the occurrence of false positive doesn’t affect the detection of highly toxic isolates by ammonia vapour test, which is the underlying cause of this study. Similarly, Saito and Machida [20] noted a low frequency of false-positives and false-negatives (4% and 10%, respectively) in *Aspergillus* isolates on exposure with ammonia vapour. The detection of 28.5% of false negative in pink category signifies that the aflatoxin producing ability of isolates estimated by ELISA falls in 21-500ppb category but the corresponding colour of ammonia vapour test didn’t coincides with the respective category *i*.*e*., pink colour. However the appearance of false negatives doesn’t affect the identification of highly toxic isolates. The 88.5% agreement between ammonia vapour test and ELISA in cream colour category highlights the sensitivity of the test in identifying the low concentrated isolates (Table 2). The 100% agreement in high toxic categories reinforces our study to identify the highly toxic isolates from bulk sample of *Aspergillus flavus* isolates with ease. This method is simple, require less expertise, time saving and inexpensive as with one litre of ammonia solution approximately 500 samples can be screened effectively for aflatoxin producing fungi from maize food/ feed bulks.

**Table 2:** Agreement between ammonia vapour test (cultural method), TLC with enzyme—linked immunosorbent assay (ELISA) in detection of atoxigenic/ toxigenic *A*. *flavus* isolates.

Categories	Agreement with ELISA			
	AFB1 conc. (ppb)^a^		Ammonia Vapour Test (%)	TLC (%)
**Cream (least/atoxic)**	<20	Agreement	88.50	84.60
		False Positive	11.50	15.40
**Pink (moderate toxic)**	21–500	Agreement	71.5	85.7
		False Negative	28.5	14.3
**Red (high toxic)**	501–2000	Agreement	100	100
		False Negative	0	0
**Plum red (very high toxic)**	>2000	Agreement	100	100
		False Negative	0	0

^a^ AFB_1_ concentration detected by ELISA

### Validation of toxigenic behaviour of *A*. *flavus* isolates in maize kernel

In cultures, *A*. *flavus* isolates showed variability in toxin producing ability (Table 2). To validate their different toxin producing behaviour in maize grains, the maize hybrid (Vivek QPM 9) inoculated with *A*. *flavus* isolates from four aflatoxin concentration categories *i*.*e*., highly toxic isolate (AF22), toxic isolate (AF-14), moderately toxic isolate (AF-35) and least toxic isolate (AF-28). To estimate the aflatoxin build-up in the grains two control were used. First control was unsterilized grain sample and other one was surface sterilized with 2% sodium hypochlorite solution. In surface sterilized check5.73 ppb AFB_1_concentration detected. The non-sterilized check grains showed 13.35 ppb of AFB_1_ concentration which may be due to the presence of some seed borne field fungi associated with maize grains. The maize grain inoculated with highly toxic isolate (AF-22) produced maximum concentration of AFB_1_ *i*.*e*., 1021.78 ppb. While the toxic (AF-14), moderately toxic (AF-35) and least toxic (AF-28) isolates exhibited 807.10, 577.89, and 11.67 ppb AFB_1_ concentration, respectively (Table 3). This experiment validates the similar behaviour of toxin producing character of *A*. *flavus* isolates in, *in-vivo* and *in-vitro* conditions. The 100% agreement of colorimetric visualization test (Ammonia Vapour test) with ELISA and TLC and further validation in maize grains in respect of highly toxic and toxic isolates, strengthen the use of ammonia vapour test as pre-screening method for segregating contaminated samples from bulk stock without the need of chemical extraction (ELISA and TLC, expense and infrastructure.

**Table 3:** *In vivo* toxigenic behaviour of *A*. *flavus* isolates from four aflatoxin categories.

In-vivo conditions	Isolates	Aflatoxin concentration ^a^ (ppb)
**Control (sterilized sample)**	No isolate	5.73±0.08
**Initial (unsterilized sample)**	No isolate	13.35±0.01
**Least toxic**	AF-28	11.67±0.73
**Moderately toxic**	AF-35	577.89±16.37
**Toxic**	AF-14	807.10±20.5
**Highly toxic**	AF-22	1021.78±61.51

^a^ Mean±S.D.

No. of replications = 3

## Conclusion

The study demonstrates the efficiency of qualitative method *i*.*e*., ammonia vapour test for detection of aflatoxigenic fungi in maize. The analytical methods (TLC and ELISA) which are accurate and reliable, requiring large quantity of expensive organics with expertise showed 100% agreement with simple and cheaper ammonia vapour test for identification of highly toxic and toxic isolates of *Aspergillus flavus*. Also, in the study the agreement of toxin producing behaviour of *A*. *flavus* isolates by ELISA and ammonia vapour test in four different toxic categories has been conclusively validated in maize grains. With this amount of accuracy, ammonia vapour technique can be advantageous to pre-screen the grain samples contaminated with *A*. *flavus*, without the need of equipments and chemical extraction. Hence, the ammonia vapour test can be explored as easy, time and manpower saving method for segregation of most contaminated samples from bulk samples of food/feed.

## Supporting information

S1 Dataset Toxicity level of *A*. *flavus* isolates from different maize growing areas. (PDF)

S2 Dataset Validation of toxigenic behaviour of *A*. *flavus* isolates in maize kernel. (PDF)
