# Supplementary material for: Comparative study of qualitative and quantitative methods to determine toxicity level of Aspergillus flavus isolates in maize
Source: PLoS One. 2017 Dec 15;12(12):e0189760. doi: 10.1371/journal.pone.0189760 (PMC5731729; doi:10.1371/journal.pone.0189760)
Supplement: S1 Dataset — (PDF) [file pone.0189760.s001.pdf]

S1 Dataset. Toxicity level of *A. flavus* isolates from different maize growing areas

Isolate of *A. flavus*	Place	ng/ml (ppb)
AF-1	Karnal	1539.9265
AF-2	Karnal	15.3993
AF-3	Karnal	5084.5205
AF-4	Karnal	119.7085
AF-5	Karnal	0.4766
AF-6	Karnal	78.6933
AF-7	Karnal	0.4247
AF-10	Karnal	29.8538
AF-11	Karnal	6878.5991
AF-12	Karnal	4531.5836
AF-13	Karnal	1.5735
AF-15	Karnal	2238.7211
AF-16	Karnal	0.4309

Isolate of *A. flavus*	Place	ng/ml (ppb)
AF-8	Delhi	7.3388
AF-14	Delhi	3254.6178
AF-18	Delhi	174.0304
AF-19	Delhi	3325.6382
AF-20	Delhi	4904.8418
AF-21	Delhi	23.3749
AF-22	Delhi	8116.616
AF-23	Delhi	4834.7609
AF-24	Delhi	1090.1845
AF-25	Delhi	1.0366
AF-26	Delhi	1029.2005
AF-29	Delhi	1.4538
AF-50	Delhi	3072.5574

Isolate of *A. flavus*	Place	ng/ml (ppb)
AF-27	Begusarai	29.2163
AF-28	Begusarai	0.5705
AF-30	Begusarai	3208.1155
AF-31	Begusarai	8.4747
AF-32	Begusarai	3.0947
AF-33	Begusarai	4.4668
AF-34	Begusarai	4387.2007
AF-35	Begusarai	530.8844
AF-36	Begusarai	0.2144
AF-37	Begusarai	3.9811
AF-39	Begusarai	0.2839
AF-40	Begusarai	0.2964
AF-41	Begusarai	53.4718
AF-42	Begusarai	0.7886
AF-43	Begusarai	2.4939
AF-44	Begusarai	10.0722
AF-46	Begusarai	12.0573
AF-47	Begusarai	0.9239
AF-48	Begusarai	0.5705
AF-49	Begusarai	1412.5375

Isolate of *A. flavus*	Place	ng/ml (ppb)
AF-38	Hyderabad	0.5347
AF-45	Hyderabad	8.2343

Isolate of *A. flavus*	Place	ng/ml (ppb)
AF-9	Dhaulakuan	0.2778
AF-17	Dhaulakuan	0.9107
