# Supplementary material for: Comparative study of qualitative and quantitative methods to determine toxicity level of Aspergillus flavus isolates in maize
Source: PLoS One. 2017 Dec 15;12(12):e0189760. doi: 10.1371/journal.pone.0189760 (PMC5731729; doi:10.1371/journal.pone.0189760)
Supplement: S2 Dataset — (PDF) [file pone.0189760.s002.pdf]

S2 Dataset. Validation of toxigenic behaviour of A. flavus isolates in maize kernel

S.No	Sample Name	Aflatoxin Conc. (ppb)
1	(**Control**) R1	5.693443
2	(**Control**) R2	5.840182
3	(**Control**) R3	5.684892
	**Average**	**5.739506**
4	*A. flavus* (**Initial**) R1	13.33974
	A. flavus (**Initial**) R2	13.35314
	A. flavus (**Initial**) R3	13.36938
	**Average**	**13.35408**
5	*A.flavus*- 28 (**AT**) R1	12.04073
6	*A.flavus* - 28 (**AT**) R2	12.15408
7	*A.flavus*- 28 (**AT**) R3	10.83234
	**Average**	**11.67572**
8	*A.flavus*- 35 (**MT**) R1	596.8062
9	*A.flavus*- 35 (**MT**) R2	568.2166
10	*A.flavus*- 35 (**MT**) R3	568.671
	**Average**	**577.8979**
11	*A.flavus*- 14 (**T**) R1	785.4366
12	*A.flavus*- 14 (**T**) R2	809.7793
13	*A.flavus*- 14 (**T**) R3	826.0918
	**Average**	**807.1026**
14	*A.flavus*- 22 (**HT**) R1	1083.103
15	*A.flavus*- 22 (**HT**) R2	1022.155
16	*A.flavus*- 22 (**HT**) R3	960.0792
	**Average**	**1021.779**
